# Instantaneous Conventions: The Emergence of Flexible Communicative Signals

**DOI:** 10.1177/0956797616661199

**Published:** 2016-10-28

**Authors:** Jennifer Misyak, Takao Noguchi, Nick Chater

**Affiliations:** 1 Behavioural Science Group, Warwick Business School, University of Warwick; 2 Department of Psychology, University of Warwick; 3 Department of Experimental Psychology, University College London

**Keywords:** human communication, conventions, common ground, pragmatics, coordination, context, open data

## Abstract

Humans can communicate even with few existing conventions in common (e.g., when they lack a shared language). We explored what makes this phenomenon possible with a nonlinguistic experimental task requiring participants to coordinate toward a common goal. We observed participants creating new communicative conventions using the most minimal possible signals. These conventions, furthermore, changed on a trial-by-trial basis in response to shared environmental and task constraints. Strikingly, as a result, signals of the same form successfully conveyed contradictory messages from trial to trial. Such behavior is evidence for the involvement of what we term *joint inference*, in which social interactants spontaneously infer the most sensible communicative convention in light of the common ground between them. Joint inference may help to elucidate how communicative conventions emerge instantaneously and how they are modified and reshaped into the elaborate systems of conventions involved in human communication, including natural languages.

The psychology of human communication has primarily focused on how people convey messages to each other using elaborate systems of conventions, including language, facial expressions, and gestures. But people can also communicate surprisingly effectively even with few conventions in common—when they have to jointly create communicative conventions on the spot. We call these *instantaneous conventions*. Vivid examples of this ability are recorded in stories of first contact between different cultures. For example, on encountering the explorer James Cook, two Fuegian islanders stepped forward to display and then throw aside large sticks, gestures that Cook interpreted as indicating peaceful intentions. Indeed, the Fuegians and Cook were soon exchanging gifts and eating together on board ship (Hough, 1994). This ability also appears to be at work when pidgins naturally develop between adult communities lacking a common linguistic background (e.g., the 18th-century creation of Russenorsk by Russian traders and Norwegian fisherman to facilitate seasonal bartering; Jahr, 1996) and in deaf children’s invention of home-sign gestures to communicate with hearing caregivers (Goldin-Meadow & Feldman, 1977). More prosaically, young children without a common language can, nonetheless, communicate well enough for complex social play (Protassova, 1992).

How is such communicative kick-starting possible? We explored the creation of instantaneous conventions using an experimental paradigm involving a common task, a simplified environment, and the most minimal possible communicative signals. A natural intuition about experimental studies of spontaneous conventions is that new conventions will be shaped by preexisting conventions or that new, stable conventions will rapidly form during the experiment. A critical test for the instantaneous nature of conventions observed in our paradigm, then, is that they should flexibly change to fit the communicative constraint of the moment. Ideally, the same signal might successfully convey contradictory messages from trial to trial, depending on task and environmental constraints.

We drew on Lewis’s (1969) legacy of analyzing signaling games to elucidate principles of communication. Recently, laboratory experimental-semiotics games (e.g., Galantucci, 2005; Scott-Phillips, Kirby, & Ritchie, 2009; for a review, see Galantucci & Garrod, 2011) have helped researchers explore the early emergence of signaling conventions among paired or grouped participants over successive interactions. Many such studies employ Pictionary-style tasks, in which participants initially produce iconic representations of meanings; over time, these signals evolve into new graphical signs with different semiotic properties (e.g., becoming simpler and more symbolic; Fay, Ellison, & Garrod, 2014). In a complementary paradigm, iterated-learning experiments focus on how structural design features of language may emerge from prespecified and arbitrary signal-meaning mappings, which have been subsequently shaped by transmission through chains of individual learners (e.g., Kirby, Cornish, & Smith, 2008; Verhoef, Kirby, & de Boer, 2015; for a review, see Tamariz & Kirby, 2016). These important discoveries show how conventions may be iteratively built up across time through communicative interaction or cultural transmission. By contrast, we focused on how conventions may be instantaneously created in the moment—without any conventionalized, iconically based, or prespecified mappings from which to bootstrap them.

The same processes underpinning instantaneous conventions may also help explain how established conventions can be used with great flexibility (e.g., Clark, 1996; Grice, 1975). For instance, conversational partners jointly coordinate and adapt lexical conventions when referring to novel objects (Clark & Wilkes-Gibbs, 1986), and such choices may be shaped and reshaped over successive interactions (Brennan & Clark, 1996; see also Clark & Brennan, 1991). Over time, initially flexible communicative conventions may become increasingly entrenched, both during acquisition and use and through generations of cultural evolution.

Before considering such implications, however, we introduce our paradigm for exploring instantaneous conventions and whether people can successfully “flip” a signal’s conventional meaning in light of changing communicative and task constraints.

## Experiment 1: Creation of Signaling Conventions in a Novel Paradigm

Our initial experiment tested whether spontaneous conventions could be created and adapted in an experimental task in which adult participants could not use any preexisting languages, familiar gestures, iconic signs, established symbols, or other standard communicative systems to coordinate toward a goal.

### Method

#### Participants

Twenty-four undergraduates from the University of Warwick—with self-reported normal or corrected-to-normal vision, normal hearing, and good fluency in English (or better)—were recruited through an online participant-recruitment panel. We recruited for 24 spaces at the group session to ensure that there would be 18 participants in attendance, which we projected would be sufficient for a strong predicted effect. Two participants were dismissed partway through performing the experimental task because of problems in reestablishing a network connection; their data were excluded from analyses. (These 2 participants observed only 8 of the 64 experimental trials.) After a corresponding delay, the remaining 22 participants (10 female, 12 male; mean age = 20.6 years, *SD* = 1.8) resumed and completed the task. All participants were paid £10.

#### Communication paradigm

**Fig. 1.**
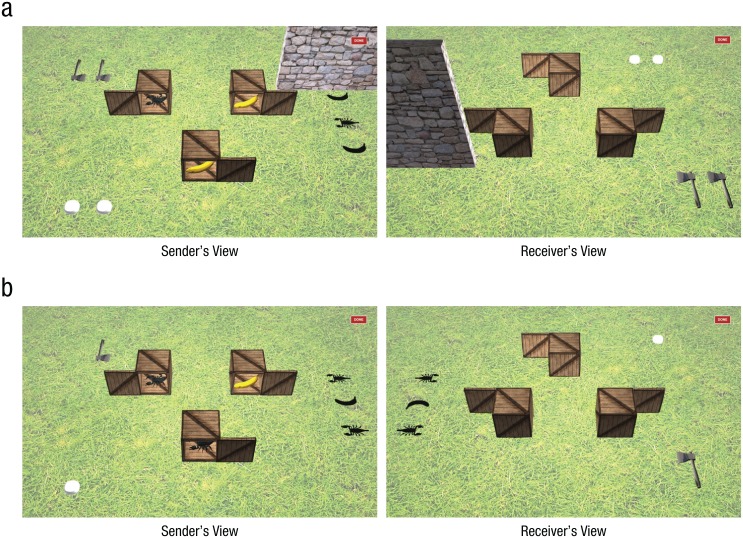
Examples of two visual scenes (a, b) from the communication game. Senders and receivers saw the same scenes but from opposite points of view. From their respective views, each player could apprehend which aspects of a given scene should be visible to his or her partner, as well as which objects were mutually visible to them both. Only senders could see the contents of each of the three boxes (a scorpion or a banana, which varied from trial to trial); however, a set of shadows on the ground matched the quantities and types of objects inside the boxes. Though always visible to senders, these shadows were (a) sometimes occluded from receivers’ view by a wall and (b) sometimes not. Other objects in the scenes—tokens and axes—varied in number depending on the trial type (see the Method for Experiment 1 for more information about these items), and a “DONE” button appeared in the upper right corner. For purposes of illustration, the smaller-scale objects shown here have been spaced closer together and enlarged (and the grass has been lightened) for clarity.

We developed an interactive two-player computer game in which both partners viewed a 3-D-simulated scene, but each saw the scene from the opposite visual perspective (illustrated in Fig. 1). The game environment consisted of three boxes, each containing either a reward (banana) or nonreward (scorpion). The number of rewards and the rewards’ locations in the boxes, as well as other scene variables, changed from trial to trial. One partner played the role of sender, and the other played as a receiver: They shared the joint task of uncovering as many rewards as possible while avoiding nonrewards.

Contents of the boxes were visible only to the sender by means of panels that slid open on the side of the box facing the sender (see Fig. 1). However, a set of shadows (impressions of bananas and scorpions, embedded in the virtual ground) was sometimes mutually visible to both players (e.g., as in Fig. 1b). The shape and number of these shadows corresponded to the number of scorpions and bananas inside the three boxes on that trial. On other trials, however, a wall prevented the receiver from seeing these shadows (as in Fig. 1a). The presence or absence of the wall occluding the shadows thus served as a manipulation of the *common ground* between sender and receiver as to the quantities of rewards within the scene. We expected participants to be sensitive to this manipulation, although referential communication studies (Hanna, Tanenhaus, & Trueswell, 2003; Keysar, Bar, Balin, & Brauner, 2000) suggest that this kind of provisional occluder may sometimes increase the relative difficulty of trials, owing to factors in effectively differentiating between what is *privileged ground* (privately known) and common ground.

Although senders had the advantage of seeing inside boxes, only receivers were able to choose which boxes to open. Successfully accomplishing the joint goal of uncovering the most bananas, therefore, required the sender to produce some signal conveying the rewards’ whereabouts to the receiver, who interpreted the signal when choosing boxes on behalf of the pair. To this end, the sender manipulated a set of tokens (white disc-shaped objects) and the receiver manipulated a set of axes with a computer mouse. The sender went first in a trial and could move any or all tokens onto the top of any of the boxes. When the sender was finished placing tokens, he or she clicked a “DONE” button to update the receiver’s view of the scene. Observing where the sender placed tokens, the receiver then placed a single ax onto each box that he or she chose to have opened. (As only one ax could be used per box, the number of axes in the scene also constrained the number of boxes that could be chosen.) When the receiver clicked “DONE,” any chosen boxes were then axed, which revealed their contents to both players.

On any given trial, the sender had the option not to move tokens, and the receiver had the option not to select boxes. They might exercise these options if, for example, they considered a potential or transmitted signal to be too unreliable or ambiguous. When this occurred, the following text message appeared on their partner’s screen: “Your partner did not place anything.” This happened infrequently.

**Fig. 2.**
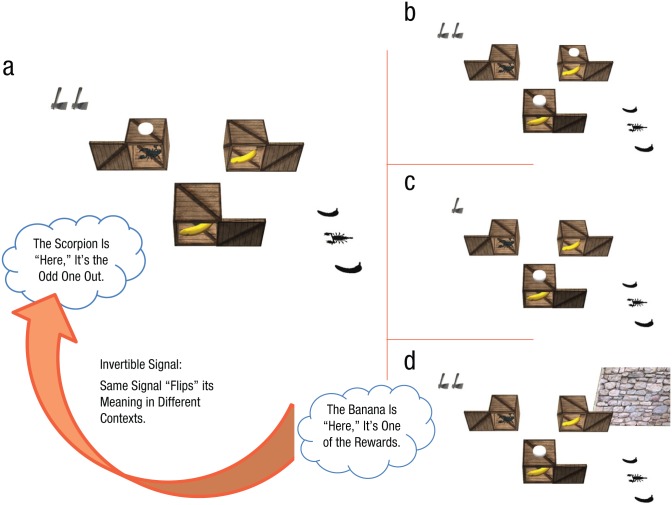
Illustration of the main elements of four key scenes (from the sender’s view), along with hypothesized placement of tokens as signals. The contents of the three boxes—two bananas and one scorpion—are identical across all four scenes. However, each of the scenes in the right-hand panels varies from the critical test scene (a) on precisely one dimension, namely the (b) number of tokens, (c) number of axes, or (d) presence of a wall. We predicted that contradictory meanings could be mapped onto the same signal (e.g., the placement of a solitary token) if participants flexibly changed conventions in light of these scene variations. We expected this to be the case for the comparison of signals between scene (a) and the other three scenes, because these scene variations change partners’ common ground either for aspects of the scene or for task constraints. That is, between (a) and (d), the wall manipulation changes whether or not it is common ground that the shadows are perceptually accessible to both players. The other scenes alter common ground for two forms of task constraints: whether the signal can be composed of one or two tokens—compare (a) and (b)—and whether the receiver can select one or two boxes (as indicated by the number of axes)—compare (a) and (c). If participants’ signaling is spontaneously responsive to these changes, then players may coordinate on the most sensible convention that is adapted to their common ground in the new situation. Consequently, the same signal can “flip” or invert its meaning, depending on communicative context.

An intuitively appealing convention for mapping signals to meanings in this communication paradigm may be to place tokens on the boxes containing rewards (as depicted in Fig. 2b).^1^ Given the appeal of this convention, would participants change it flexibly—even reversing the meaning from that attached to a previous signal—in light of a potentially better mapping in different contexts?

We reasoned that if senders were provided with only one token on a trial with two bananas, then a more advantageous mapping might be to place the token on the box containing the scorpion (i.e., to “mark,” by this token placement, the box that has the scorpion)—in contradiction to “marking” one of the boxes containing a banana. Adoption of this convention would allow players to obtain both bananas rather than just one. Crucially, such an “odd-one-out” signal would be sensible in situations in which the elements of the scene supported this inference by both players—for instance, in a situation in which it could be commonly known that there were two bananas present (as indicated by the shadows) and that the sender was limited to one token (cf. Figs. 2a and 2d). We took such a case to be a critical test of whether conventions spontaneously changed in our experiment.

Similarly, conventions might also be sensitive to restrictions on the means to attaining the common goal. For instance, the conditions outlined in the previous paragraph for the hypothesized odd-one-out signal might be fulfilled, but it may also be part of common ground that the receiver has only one ax (see Fig. 2c)—and thus, the pair is limited to obtaining only one banana. Such circumstances make the odd-one-out signal unnecessary for maximal success, which could result in the signal of a token’s placement to flip its meaning again from along the lines of “this is the odd-one-out item” to “this is one of the rewards” (cf. Figs. 2c and 2a). Such an inversion would again be indicative of spontaneous conventions in lieu of transferring the odd-one-out signaling approach to this new context.

The experiment was therefore designed to explore whether such invertible signals would emerge across these hypothetical scenarios, as depicted in Figure 2. The following variables were manipulated: tokens (one or two present), rewards (one or two present), axes (one or two present), and wall (present or absent). The wall variable manipulated whether both players had the same information about reward quantities—this knowledge was assumed to be common ground between players only when the wall was absent (thus affording both players a nonoccluded view of the shadows). Crossing all factors yielded 16 combinations (2 × 2 × 2 × 2) for specifying the composition of a game scene. Each possibility thus constituted a unique trial type, which was presented an equal number of times. Locations of rewards and nonrewards were randomized per trial across the three boxes, as were the ordering of reward and nonreward shadows.

The specifications for the subset of trial types of key interest (corresponding to the four scenes in Fig. 2) are summarized in Table 1. By presenting all of the 16 trial types an equal number of times, it was equally likely for a scene to contain one or two rewards (this was true for trials in general, as well as for the subset of trials on which the wall appeared). This fact supported the effectiveness of the wall manipulation since it precluded the receiver from otherwise exploiting probabilistic information about reward quantities whenever the wall was present.

**Table 1.** Definition of the Four Key Trial Types

Trial type	Number of tokens	Number of rewards	Number of axes	Wall
Two token	2	2	2	Absent
Inversion	1	2	2	Absent
Wall	1	2	2	Present
One ax	1	2	1	Absent

Note: When the wall was absent, the number of rewards was not only known by both partners (individually)—but the manner in which the shadows were mutually visible allowed partners to apprehend that they shared this information (jointly) as common ground. When the wall was present, partners lacked common ground for such knowledge about reward quantity. Irrespective of the wall variable, however, the number of tokens and number of axes were always perceptually evident as common ground between partners.

#### Procedure

Participants played the communication game over networked computers in the laboratory during the same group session. They first received instructions that clarified the joint goal of the interaction (collecting as many bananas as possible while avoiding scorpions), the turn-taking structure (sender goes first, then receiver), procedural details (e.g., random matching of new partners per trial, clicking the “DONE” button), the fact that the shadows indicate the quantities of rewards, and the scene objects (tokens, axes) that can be manipulated by each participant according to their role (sender, receiver). Participants then completed four practice trials corresponding to two different scene configurations. (These scenarios were simple cases involving one token, one banana, and two axes—but differed in that one scenario did not include the wall, whereas the other did.) Each scene configuration was presented twice, as two trials, to provide participants with experience of the same scene as both a sender and a receiver.

Participants completed 64 trials. Each participant was assigned to be a sender or receiver for the first half of the trials and was assigned the opposite role for the second half. Within each half, each of the 16 trial types was presented twice in a random order. A new random matching of senders and receivers was generated on each trial, which resulted in each participant anonymously interacting with several different players across the entirety of the game rather than with a fixed partner. Interaction therefore transpired both between paired participants and within a communicative “community” (i.e., a closed group of interacting participants). After completing a trial, each sender-receiver dyad watched as the chosen boxes were axed open, which revealed their contents.

### Results

Participants’ actions were coded into signaling categories. For the sender analyses, we refer to participants as having marked the particular content of a box (a reward or nonreward) if they marked that box itself (by placing a token on top of it). Accordingly, sender categories reflected whether participants marked all and only rewards (i.e., bananas), one of two rewards, or all and only nonrewards (i.e., scorpions) or whether they took some other action (e.g., not marking any boxes). The categories for receivers reflected whether they selected all and only marked boxes, one of two marked boxes (if applicable), all and only unmarked boxes (if applicable), or one of two unmarked boxes or whether they took some other action (including no selection). For each trial type and with respect to each participant role, the proportion of each participant’s actions that belonged to a signaling category was used to derive the corresponding mean proportion of the group.

For the various trial types in which there was only one banana and one token (i.e., 4 out of the 16 trial types), participants consistently placed the token on the box containing the banana (mean proportions ranged from .95, *SE* = .03, 95% confidence interval, or CI = [.89, 1.00], to 1.00, *SE* = .00). Having established that marking the reward was the clear tendency in these basic cases, we focused our analyses on the subset of trials (see Table 1 and Fig. 2)—all involving two rewards—that critically tested whether senders’ signaling patterns were flexible across other contexts. First, it was found that the same general strategy of marking rewards was also predominant on two-token trials; the proportion of such trials on which senders marked both of the boxes containing bananas was near ceiling (*M* = .98, *SE* = .02, 95% CI = [.93, 1.00]). On inversion trials, which involved the same specification of scene variables except that there was only one token available, senders’ behavior switched strongly to marking the box with the scorpion (*M* = .91, *SE* = .04, 95% CI = [.82, 1.00]).

This scorpion-marking behavior, however, was moderated by the manipulation of common ground concerning reward quantities on wall trials. When the wall precluded receivers from knowing how many bananas there were, senders on wall trials were distinctly less inclined to mark the scorpion (*M* = .48, *SE* = .07, 95% CI = [.33, .62]), sometimes marking one of the bananas instead (*M =* .50, *SE* = .07, 95% CI = [.35, .64]). Finally, on one-ax trials, which were the same as inversion trials in all respects save for the presence of one ax instead of two, senders again modified their signaling, marking one of the bananas most of the time (*M* = .68, *SE* = .08, 95% CI = [.52, .84]) instead of marking the scorpion (*M* = .32, *SE* = .08, 95% CI = [.16, .48]).

**Fig. 3.**
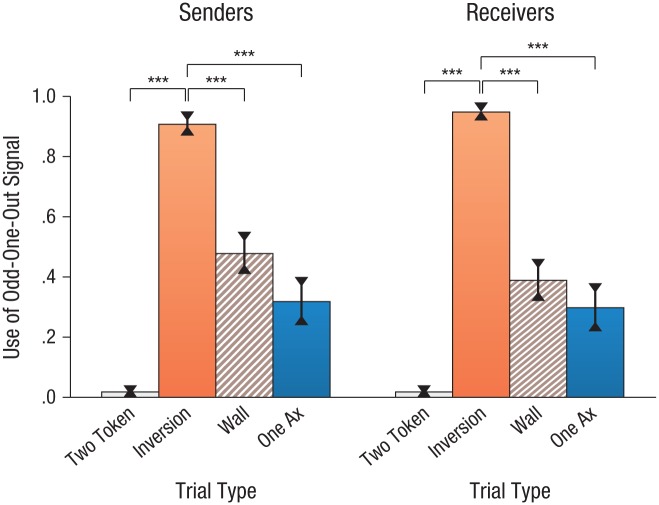
Mean proportion of trials on which participants used the odd-one-out signal in Experiment 1 as a function of trial type, separately for the sender and receiver roles. Asterisks indicate significant differences between the critical trial type (inversion) and other trial types (*p* < .0001). Error bars represent ±1 *SE*.

To directly compare signaling across these key trial contexts, we analyzed participants’ use of the odd-one-out signal (see Fig. 3). For senders, use of an odd-one-out signal was defined as marking the box containing the scorpion; for receivers, use of an odd-one-out signal was defined as selecting both of the unmarked boxes (or selecting one unmarked box when limited to only one ax). Senders’ proportions of odd-one-out signals were strongly affected by trial type, as indicated by a one-way repeated measures analysis of variance (ANOVA), *F*(2.31, 48.49) = 44.82, *p* < .001, η_*p*_^2^ = .68. Planned comparisons revealed that, compared with senders’ ubiquitous use of the odd-one-out signal on inversion trials, senders’ use of odd-one-out signals on the other key trial types was sharply reduced—difference between inversion and two-token trials: *M* = .89, 95% CI = [.79, .98], *p* < .001; between inversion and wall trials: *M* = .43, 95% CI = [.26, .60], *p* < .001; and between inversion and one-ax trials: *M* = .59, 95% CI = [.40, .78], *p* < .001. A similar significant pattern of findings was seen for receivers (Fig. 3), which reflects congruency in signaling approaches for both interactional roles. We stress that the odd-one-out signal was the most common response even on the very first relevant trial, both for senders and receivers, and that this level of responding did not differ significantly with that for later trials. Thus, this flip of the interpretation of the signal arose instantaneously rather than building up slowly across trials.

### Discussion

Participants generated shared signaling conventions, including the instantaneous emergence of the odd-one-out signal of marking the scorpion. Comparisons indicated that the selectively strong use of this signal was not merely a response to the scenario with two bananas and one token. Odd-one-out signals did flexibly appear when communicative constraints changed from two available tokens to one—but also crucially depended on whether the scene permitted as common ground that there were two rewards. Without this common ground, the odd-one-out convention was curtailed. Moreover, players demonstrated sensitivity to common ground concerning not only rewards but also available actions: The presence of a single ax indicated that the receiver could open only one box, so sender and receiver flipped their signaling approach back to marking the banana. This critical role of common ground is consistent with its prominence in theoretical accounts of coordination (e.g., Schelling, 1960), conventions (e.g., Lewis, 1969), and language (e.g., Clark, 1996).

In sum, across the four key trial types, participants created and used invertible signals, felicitously reversing the prior meanings of signals of the same form. The implications of this finding will be considered in the General Discussion.

## Experiment 2: Signaling Under Asynchronous Conditions

While Experiment 1 explored signaling under conditions concomitant with real-time communication, Experiment 2 investigated whether invertible signals appear under stringently controlled conditions that preclude interactive or outcome-based feedback. The design further controlled for potential ordering effects that could stem from participants experiencing the same sequence of randomly ordered trials.

### Method

#### Participants

Two separate groups of 16 participants each were needed to instantiate our counterbalanced design (see the following section). To ensure this number of participants would be in attendance, we recruited from an online participant panel to fill 21 spaces for each of two sessions. Recruits were advised before signing up that sessions required a particular configuration of participants, and, therefore, there was a possibility that some recruits might be dismissed at their session’s start with £3 compensation.

Six recruits did not attend their session, which left 4 surplus recruits in attendance (3 in the first session, 1 in the second session); accordingly, 4 recruits were randomly selected and dismissed with £3 payment. The remaining 32 participants were all undergraduate students (15 male, 17 female; mean age = 20.3 years, *SD* = 1.9) from the University of Warwick. All self-reported having normal or corrected-to-normal vision, normal hearing, and good fluency in English (or better). Each participant was paid £7 for participating in one of the two sessions (which corresponded to different conditions, as described in the following section). None of the participants had taken part in Experiment 1.

#### Design and procedure

The interactive components of the communication game from Experiment 1 were removed, and the task was decomposed into two parts: (a) a sender condition in which trials consisted only of viewing game scenes (from the sender’s view) and generating signals and (b) a receiver condition in which trials consisted only of viewing signals embedded in game scenes (from the receiver’s view) and selecting boxes.

The same 16 trial types from Experiment 1 were presented across four blocks, with each trial type presented once per block. Four 16 × 16 Latin squares were used to specify 16 unique sequences of 64 trials each to be assigned, for each condition, across participants. Each Latin square counterbalanced ordinal position, immediate sequential effects, and remote (second-, third-, fourth-order, etc.) sequential effects of the 16 trial types within each block. Care was also taken to ensure balancing for first- and second-order sequential effects introduced by the transitions of trials between blocks and to avoid immediate repetitions or alternations of the same trial type at the end of one block and the beginning of the next. (More details on the construction of these Latin squares, as well as a table of the full sequences from the experiment, are provided in the Supplemental Material available online.)

Participants in the sender condition generated all the signals that were used for those in the receiver condition. The identical set of trial sequences (specified by the Latin squares) for the sender condition was also administered in the receiver condition. Off-line, a matching algorithm randomly assigned senders’ signals to receivers, with the constraints of limiting the frequency of pairing with a given sender—per block (≤ 3) and overall (≤ 8)—and the number of signals (≤ 3) from a given sender for the same ordinal trial (across participants). This method assigned signals for interpretation in the same relative time frame as they were produced (i.e., signals generated earlier were interpreted early in the session). It also allowed receivers to receive input from multiple random partners (as in Experiment 1), while preventing any particular sender’s signals from being unduly represented across a time slice.

The two conditions (sender, receiver) were conducted on different days. The instructions were similar to those given in Experiment 1 but modified to account for the new design. Participants also completed the same practice trials (in the same manner) as in Experiment 1 to familiarize them with the differing visual perspectives of each role. We specifically mentioned to participants that the number of axes was unrelated to the number of rewards.

The communication game was then played without live interaction. Participants in the sender condition generated all the signals that were later presented to participants in the receiver condition. In their session, receivers interpreted the signals previously generated by the senders. No feedback on outcomes (i.e., the contents of axed boxes) was provided to the receivers (or, necessarily so, to the senders).

### Results

Participants’ actions were coded into the same signaling categories as in Experiment 1. Within each condition (sender, receiver), the mean proportion of participants’ actions belonging to a signaling category was calculated for each trial type.

We focused our analyses on the same key trial types as in Experiment 1. On two-token trials, senders always marked the two boxes containing bananas (*M* = 1.00, *SE* = .0). On inversion trials, senders marked the scorpion a substantial proportion of times (*M* = .56, *SE* = .10, 95% CI = [.34, .78]), although, at other times, they marked one of the two boxes containing a banana (*M* = .42, *SE* = .10, 95% CI = [.20, .64]). On wall trials, senders strongly favored marking one of the two boxes containing a banana (*M* = .77, *SE* = .08, 95% CI = [.60, .93]), as opposed to marking the box containing the scorpion (*M* = .23, *SE* = .08, 95% CI = [.07, .40]). And on one-ax trials, senders chose to mark one of the bananas fairly often (*M* = .63, *SE* = .10, 95% CI = [.41, .84], marking the scorpion on the remainder of occasions (*M* = .38, *SE* = .10, 95% CI = [.16, .59]).

**Fig. 4.**
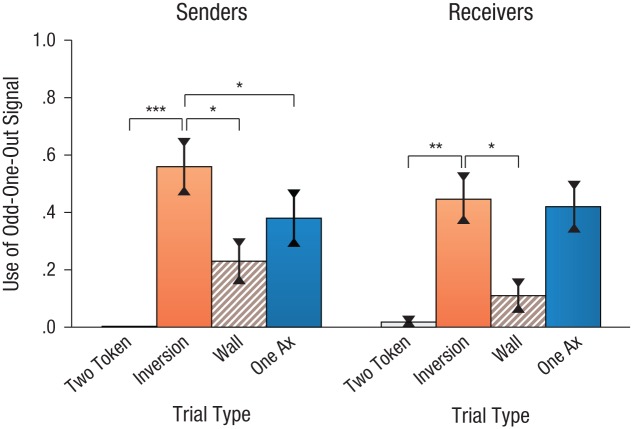
Mean proportion of trials on which participants used the odd-one-out signal in Experiment 2 as a function of trial type, separately for the sender and receiver conditions. Asterisks indicate significant differences between the critical trial type (inversion) and other trial types (*p < .01, **p < .001, ***p < .0001). Error bars represent ±1 *SE*.

Figure 4 displays the mean proportion of trials on which senders and receivers used the odd-one-out signal. Senders’ odd-one-out signals were strongly affected by trial type, as indicated by a one-way repeated measures ANOVA, *F*(2.01, 30.11) = 12.20, *p* < .001, η_*p*_^2^ = .45. Planned comparisons revealed that senders used odd-one-out signaling less often on two-token, wall, and one-ax trials than on inversion trials (mean difference between inversion and two-token trials = .56, 95% CI = [.34, .78], *p* < .001; between inversion and wall trials = .32, 95% CI = [.11, .55], *p* = .007; and between inversion and one-ax trials = .19, 95% CI = [.05, .32], *p* = .009).

A generally similar pattern was seen for receivers, although the use of the odd-one-out signal on the inversion trials was nominally lower (*M* = .45, *SE* = .09, 95% CI = [.25, .65]) than for senders, despite being nominally preferred over marking one of the bananas (*M* = .33, *SE* = .08, 95% CI = [.15, .50]). In the absence of feedback or interactivity, a notable proportion of receivers’ actions on inversion trials consisted of alternate strategies (*M* = .22, *SE* = .08, 95% CI = [.06, .38]), which may have reflected participants’ reduced sense of engaging in a communicative situation or a hedging between the mark-the-banana and odd-one-out conventions (e.g., receivers might select only one of the unmarked boxes but not the marked box, select one marked and one unmarked box, or select nothing). Consequently, receivers’ use of the odd-one-out signal on inversion trials did not differ significantly from their use of the odd-one-out signal on one-ax trials (*p* > .250), in which alternate selection strategies (*M* = .02, *SE* = .02) were only negligibly deployed.

Because the inversion trial type was so critical, we additionally note that, in both conditions, the presence of the odd-one-out convention was substantial, with about 72% of participants using the odd-one-out signal at least once on inversion trials.

### Discussion

Odd-one-out signaling was attenuated in Experiment 2 relative to Experiment 1, but it was nonetheless substantially present and was transmitted significantly more often in the appropriate context, inversion trials, than on other types of trials. Thus, invertible signals emerged, despite controls that balanced potential trial-order effects and eliminated feedback. While differences between experiments were not intended to isolate the contribution of feedback (as additional design aspects were manipulated), participants’ selective adoption of the odd-one-out convention indicates that feedback is not necessary to account for invertible signals. Instead, interaction-based feedback may plausibly amplify usage of invertible signals in more naturalistic settings.

## General Discussion

In a nonlinguistic paradigm, participants instantaneously created communicative conventions using the most minimal signals. Signals of identical form carried contradictory meanings from moment to moment, and they did so flexibly in response to the shared scene and task constraints. That is, the same signal of a placed token could signify something akin to either “here’s a banana, choose this” or “here’s a scorpion, don’t choose this” under almost identical circumstances—the same distribution of two bananas and one scorpion inside three boxes. Subtle changes in the common ground between senders and receivers, as read from visual cues in the virtual environment (axes, tokens, wall), allowed people to shift to more task-appropriate conventions (e.g., where two bananas are obtainable).

Do invertible signals arise outside our experimental task? We suggest that this phenomenon is reminiscent of contronymy^2^ (Karaman, 2008) in natural language. A *contronym* is a word with opposing meanings. English examples include the verb *dust*, which may refer to either adding or, alternatively, removing fine particles from something (e.g., “dust the cake” vs. “dust the trophy”), and the adjective *wicked*, which may describe both extremely bad or extremely good things—the latter sense documented as far back as, at least, Fitzgerald’s *This Side of Paradise* (1920, p. 105). Because different uses of a contronym are identical in word form (both orthographically and phonologically) and syntactic word class, their meanings must be distinguished by context (linguistic or situational). Invertible signals in our paradigm are analogous to contronyms with respect to having these same contradictory, context-dependent qualities. Their meaning, too, is distinguished by the context (afforded by the visual scene). Invertibility is, more broadly, an example of ambiguity in a communication signal that is resolvable by context, which will be a feature of any coding system that is optimized to transmit as much information as possible (Piantadosi, Tily, & Gibson, 2012).

The contronymlike qualities of invertible signals may also have their counterpart in some instances of verbal irony (Gibbs, 2000; Roberts & Kreuz, 1994), in which an utterance’s meaning in the moment may contradict its more typical meaning. For example, someone may say “that’s just what we need” on hearing news of a setback instead of a breakthrough.

Verbal irony and contronyms are sharp manifestations of context dependence, as the meaning of the same word or utterance may entirely flip. Less extreme shifts of meaning, depending on context, pervade natural language—as studied especially in pragmatics. Many traditional approaches in linguistics, philosophy, and psycholinguistics treat pragmatic factors as a sophisticated late stage of analysis, following after semantic and syntactic processes (e.g., as computed after the decoding of literal meaning; e.g., Fodor, 1983; Grice, 1975). Crucially, in contrast to this traditional view, pragmatic context dependency was seen here in basic communicative behavior as intrinsic to the spontaneous creation of conventions in our paradigm. Its appearance in our task supports a central and foundational role for pragmatic constraints in determining the semantic content in human communication (see Clark, 1996; Levinson, 2000; Noveck & Sperber, 2004; Sperber & Wilson, 1986).

What type of accounts might explain the establishment of conventions in our paradigm? One hypothesis, suggested for learning lexical conventions, is of a simple associative nature: Individuals learn word mappings by detecting reliable correlations between word labels and their corresponding entities in the world (Locke, 1690/1964). Current approaches of this nature in the psychological literature include associative cross-situational accounts (L. Smith & Yu, 2008; Yu & Smith, 2007), by which individuals track, across situations, co-occurrence frequencies of auditory sounds with visual percepts of objects. However, the instantaneous mapping of contradictory meanings onto the same signal would seem puzzling from an associative perspective.

Adding to the puzzle is the observation that individuals in our task could not directly apply preexisting conventions. Although their signaling behavior could arguably be seen as creating new precedents for the community, these behaviors did not become rigidly fixed for subsequent communication trials. Indeed, without any relevant reinforcement history in Experiment 2, participants still inverted signal meanings in principled ways. The genesis of these conventions would implicate, instead, the key involvement of inferential processes. It seems plausible that inferential processes may, similarly, be required in resolving anaphora (*he, it . . .*), definite descriptions (*the cup*), deictic expressions (*this, there, now, you . . .*), names, and so on, in which speakers and hearers must coordinate on one of many possible referents on the basis of their common ground (Clark, 1996).

In particular, we suggest that the present results point beyond inferential processing in general^3^ and require what we term *joint inference* (by analogy with joint action and joint attention; Scaife & Bruner, 1975; Sebanz, Bekkering, & Knoblich, 2006). That is, participants faced a coordination problem that required them to converge on the same signal-meaning mapping from a vast array of possible mappings. As a solution, participants adopted the most sensible mapping that could be *jointly inferred* from the common ground and task constraints of the moment.

By contrast, the experimentally observed conventions cannot be explained as individualistically inferred, according to what is most rational or salient from one’s own role as sender or receiver. (Otherwise, for instance, senders would have incorporated privileged information about reward quantities on wall trials.) Nor are conventions “other-regarding” solutions or predictions, as they are determined jointly from both parties’ constraints.

Notably, both parties’ joint inferences were instantaneously changing in response to the new communicative constraints of each task. The framework of joint inference accords with the perspective of viewing language and human communication as the study of joint action (Clark, 1996; Croft, 2011; Galantucci, 2009). Joint inference may be important in understanding not just how spontaneous conventions arise but how they are continually modified and reshaped in new communicative contexts, and thus how they become entrenched into increasingly rich and stable communicative systems of gestures, facial expressions, and signed and spoken natural language.

## Supplementary Material

Supplementary material

## Notes

1. Our argument does not depend on this choice of convention. The key question here is whether conventions of whatever form can change flexibly from moment to moment.

2. Contronymy is also called *enantiosemy* (Klégr, 2013).

3. For examples of productive models within the broader inferential framework, see Bergen, Levy, and Goodman (2016); Frank and Goodman (2012); and N. J. Smith, Goodman, and Frank (2013).
